# Assessing the Impact of Germination and Sporulation Conditions on the Adhesion of *Bacillus* Spores to Glass and Stainless Steel by Fluid Dynamic Gauging

**DOI:** 10.1111/1750-3841.13940

**Published:** 2017-11-10

**Authors:** Ke Xu Zhou, Nan Li, Graham Christie, D. Ian Wilson

**Affiliations:** ^1^ Dept. of Chemical Engineering and Biotechnology Univ. of Cambridge Philippa Fawcett Drive Cambridge CB3 0AS, U.K

**Keywords:** adhesion, *Bacillus*, DLVO, fluid dynamic gauging, surfaces, spore

## Abstract

**Abstract:**

The adhesion of spores of 3 *Bacillus* species with distinctive morphologies to stainless steel and borosilicate glass was studied using the fluid dynamic gauging technique. Marked differences were observed between different species of spores, and also between spores of the same species prepared under different sporulation conditions. Spores of the food‐borne pathogen *B. cereus* were demonstrated to be capable of withstanding shear stresses greater than 1500 Pa when adhered to stainless steel, in contrast to spores of *Bacillus subtilis* and *Bacillus megaterium*, which detached in response to lower shear stress. An extended DLVO model was shown to be capable of predicting the relative differences in spore adhesion between spores of different species and different culture conditions, but did not predict absolute values of force of adhesion well. Applying the model to germinating spores showed a significant reduction in adhesion force shortly after triggering germination, indicating a potential strategy to achieve enhanced removal of spores from surfaces in response to shear stress, such as during cleaning‐in‐place procedures.

**Practical Application:**

Spore‐forming bacteria are a concern to the food industry because they have the potential to cause food‐borne illness and product spoilage, while being strongly adhesive to processing surfaces and resistant to cleaning‐in‐place procedures. This work is of significance to the food processors and manufacturers because it offers insight to the properties of spore adhesion and identifies a potential strategy to facilitate the removal of spores during cleaning procedures.

## Introduction

Bacterial endospores (hereafter spores) are formed by members of the *Bacillales* and *Clostridiales* orders upon sensing nutrient depletion during vegetative growth. Spores are of major concern to the food industry because a number of species are pathogenic, whereas others are associated with spoilage. Compounding matters is the fact that spores are extremely durable, their metabolically dormant protoplast being protected by a multilayered proteinaceous coat and other features that enable them to withstand physicochemical and environmental stresses that would kill vegetative bacteria. In addition, spores of several species also have the capacity to adhere to materials of importance in the processing industries, and hence, several studies have been conducted to elucidate the mechanisms involved. Adhesion of potentially toxinogenic *Bacillus cereus* spores, for example, which presents a considerable hazard to the dairy industry given its prevalence in dairy products (Elaine and others 2011), has been studied in terms of the spore's physical properties (hydrophobicity, electrostatics, and so on; Husmark and Rönner 1992) and external morphological features that is the presence of an outermost layer of the coat referred to as the exosporium, which may also be decorated with various appendages (Faille and others 2010). Similarly, at the macro‐molecular level, the significance of proteins that comprise the outermost hair‐like nap of spores of this species has also been the subject of study (Lequette and others 2011). These studies have frequently employed multiple strains of *B. cereus*, and as such, variation in the aforementioned physical and morphological features between strains make it difficult to determine the contribution of individual factors to spore adhesion.

In terms of techniques employed to study spore adhesion, static adhesion assays have been utilized (Husmark and Rönner 1990), although flow‐based shear‐stress assays involving radial flow cells (Klavenes and others 2002) or tapered channels (Mercier‐Bonin and others 2011) are arguably more appropriate. However, many flow‐based assays used to date are associated with limitations in the shear stress that can be imposed on the test surface. This paper introduces the fluid dynamic gauging (FDG) technique to measure the adhesion of various species of spores to stainless steel and to glass. FDG, developed by Tuladhar and others (2000), has several advantageous features. For example, it can reliably impose a large range of shear stresses—from <50 to >1000 Pa—over a small area, permitting multiple measurements to be conducted on the same test sample. Also, the technique does not require large volumes of test liquid. In the mode used here the inventory of liquid is fixed, hence, the technique lends itself to contained operation, which is desirable for work with pathgenic organisms.

In addition to studying adhesion of *B. cereus* and *B. megaterium* on stainless steel and glass, the effects of sporulation conditions and the induction of germination on spore adhesion are investigated. The results are compared with forces of adhesion predicted by an extended DLVO model.

## Materials and Methods

### Bacterial strains and sporulation conditions

This study considered 3 wild‐type bacterial strains: *B. megaterium* QM B1551, provided by Prof. P.S. Vary (Ill., U.S.A.); *B. cereus* 569, provided by Prof. A. Moir (Sheffield, U.K.), and *B. subtilis* PS832, provided by Prof. P. Setlow (Conn., U.S.A.). The sporulation conditions and procedures followed were those described previously by Nicholson and Setlow (1990), Clements and Moir (1998), and Christie and others (2010) for the sporulation of *B. subtilis*, *B. cereus*, and *B. megaterium*, respectively.

For the latter 2 species, the sporulation conditions were modified in pH, salt concentration, and temperature to assess the impact of these parameters on spore adhesion. Spores were produced at 3 temperatures, namely 25, 30, and 37 °C, which were selected to represent conditions encountered in poorly managed food transport, storage, and display operations as well as biomedical cases. The range of pH conditions encountered in food processing operations is wide: for the species considered here, preliminary scoping tests indicated that adherent spores could be obtained in the pH range 4 to 9. The initial pH of the culture media was adjusted to 4.0, 7.0, or 9.0 by adding 10 M potassium hydroxide or hydrochloric acid. The total salt concentration was scaled up for each salt to a factor of 5× and 12.5× the original (Table S1). In the 1st instance, only one parameter was varied at a time. Spores were also produced under a combination of changes with the intention to minimize the adhesive properties in each species. The range of spore culture conditions used in this work are listed in Table 1. Typically, *B. cereus* spores are washed with 0.1 vol% Tween 20 surfactant to minimize spore aggregation during resuspension. To avoid altering spore surface properties, no surfactant was used in the washing stages: aggregation was avoided by carefully resuspending spores with a pipette.

**Table 1:** Spore culture conditions and their corresponding labels

		Culture conditions			
Species	Label	Initial pH	*T* (°C)	Salt	Observations
*B. megaterium* QM B1551					
	BMEG	7.2	30	Std.	
	BMEG25	7.2	25	Std.	
	BMEG37	7.2	37	Std.	
	BMEGPH9	9.0	30	Std.	
	BMEGPH5.5	5.5	30	Std.	
	BMEG1	7.2	30	×5	
	BMEG2	7.2	30	×12.5	S pores did not grow
	BMEG1,25,PH9	9.0	25	×5	
*B. cereus* 569					
	BCEREUS	7.2	30	Std.	
	BCEREUS25	7.2	25	Std.	
	BCEREUS37	7.2	37	Std.	
	BCEREUSPH9	9.0	30	Std.	
	BCEREUSPH5.5	5.5	30	Std.	
	BCEREUS1	7.2	30	×5	
	BCEREUS2	7.2	30	×12.5	
	BCEREUS2,PH9	9.0	30	×5	
*B. subtilis* PS832					
	BSUBT	7.2	37	Std.	

### Sample preparation

Circular coupons of diameter 50 mm and thickness 1 mm were used as substrates for spore adhesion. Microscopy‐grade borosilicate glass coupons were supplied by Soham Scientific (Ely, U.K.) and mirror finish stainless steel 316 coupons were obtained from Lasermaster (Redruth, U.K.). Glass coupons were immersed in a concentrated sulphuric acid solution for 15 min before being rinsed with copious amounts of distilled water and finally left to air dry in a Class II microbiological safety cabinet. Stainless steel coupons were subjected to the same cleaning procedure but immersed in isopropanol. To prepare the sample, 750 μL of a concentrated (approximately 3 wt.%) spore suspension was carefully spread over the entire surface of the coupon by tilting. This film was left to rest for 5 to 10 min to allow the spores to settle and adhere. The coupon was subsequently spun at approximately 250 rpm on a customized hard disc drive spin coater to remove any excess water and simultaneously dried with a hair drier (see Supplementary Information, Figure S1). Once the central area of the coupon had dried, as judged by eye, the remaining liquid was spun off at high speed (>2000 rpm). Each sample was inspected visually to ensure coating smoothness and uniformity and the initial coverage area, *N*_0_, was then determined by optical microscopy. Finished samples were stored in a sealed container at room temperature and subjected to gauging within 4 h of preparation.

### Fluid Dynamic Gauging

The FDG configuration used in this study is identical to that used by Salley and others (2012) to investigate the strength and structure of photosynthetic biofilms. Detailed descriptions of the operation and analysis of FDG are given by Gordon and others (2010) and Salley and others (2012). In brief, liquid is ejected from a vertical convergent nozzle located a small distance (the clearance, *h*, see Figure S2) above the test surface. The liquid flows radially away under the rim, generating a high shear stress on the surface beneath the nozzle rim. Pressure mode ejection FDG was used in this work, with deionized water at 20 °C as the gauging liquid at a flow rate of 0.8 mL/s. These conditions represented a good compromise between accuracy and range of achievable shear stresses (50 to 1500 Pa, calculated using Eq. 4, see Theory section).

Gauging tests involved setting the nozzle at a prescribed value of *h* and subjecting the layer to the above flow rate for 2 min. The nozzle was then raised, shifted to the next location, *h* adjusted and the process repeated. The gauging period was set by the capacity of the syringe pump barrels. The amount of removal is expected to depend on the time exposed to the shearing fluid and the technique could be used to investigate the kinetics: this was not attempted as part of this study.

The nozzle footprint is approximately 4*π* mm^2^ and the region of high shear stress is limited to the region beneath the nozzle rim. Between 6 and 14 locations were tested for each disc. After all locations had been tested, the disc was removed from the liquid and left to dry in air. Once dry, each gauging site was inspected with an optical microscope and 8 high magnification (400×) images were taken of each site. ImageJ (NIH, Bethesda, M.D., U.S.A.) was used to count the number of spores that remained in the gauged area, *N*.

### Contact angle measurements

Contact angles were measured by the sessile drop method using a Dataphysics OCA 15 EC contact angle goniometer. For stainless steel and glass, the liquid droplets were deposited directly onto the solid surface and the contact angles were recorded. About 40 μL of purified bacterial spore suspension, with a loading of approximately 10 wt% spores, were deposited on a clean glass substrate and dried in air for 1 h. This gave a thick, smooth lawn of spores and was used for contact angle measurements. Four liquids were used: distilled water, formamide, ethylene glycol, and (nonpolar) diiodomethane. A 1 μL droplet of liquid was pipetted onto the surface of the spore lawn and the resulting droplet behavior was captured in real time by the apparatus’ high‐speed video camera. At least 10 measurements were performed for each combination of liquid and substrate/spore species. The image sequences were processed to extract 2 data per drop, corresponding to the contact angles on the left and right sides.

### Zeta potential measurements

The zeta potential of bacterial spores was measured at pH 7.0 using a NanoBrook ZetaPALS (Brookhaven Instruments, New York). For each measurement, spore suspensions were diluted using distilled water to an OD_600_ between 0.8 and 1.0 and aliquoted into cuvettes containing approximately 3 mL of liquid. Between 8 and 10 repeat measurements were performed on each cuvette and the average of at least 10 measurements is reported for each spore sample.

### Spore imaging

Scanning electron microscopy (SEM) was conducted using an FEI Verios XHR (Oreg., U.S.A.). Approximately 20 μL of a low concentration spore suspension (OD_600_ < 1) was deposited on a circular glass cover slip and left to dry in air. Once dry, the samples were sputter coated with a layer of platinum then imaged.

### Spore germination studies

*B. megaterium* and *B. cereus* spores cultured under standard conditions were germinated to explore the effects of germination on their surface properties. Before initiating germination, a 50 mL spore suspension was heat shocked at 60 °C for 20 min to synchronize the spores for germination. After heat shocking, the spore suspension was divided into 3 equal volumes and concentrated into a pellet by centrifugation. Approximately 5 mL of LB broth was then added to each pellet and the mixture resuspended by vortexing. The containers were then placed in a shaking incubator at 30 °C. Each container was subsequently withdrawn from the incubator after different time intervals, namely 10, 50, and 110 minutes. The suspension of withdrawn germinating spores was quickly washed 3 times with distilled water and prepared for imaging, contact angle, and zeta potential measurements.

Vegetative cells were also prepared for contact angle and zeta potential measurements. Approximately 5 mL of LB broth was inoculated with *B. megaterium* or *B. cereus* spores and left to grow overnight. Vegetative cells were then collected by centrifugation and washed 5 times in distilled water before being used for further experiments. Vegetative cells were freshly grown and prepared before each experiment.

## Theory

### Fluid Dynamic Gauging

The key components of the fluid dynamic gauging device are shown in Figure S2. The nozzle and sample are immersed in the gauging liquid. The mass flow rate, *m*, and clearance, *h*, determine the pressure drop across the nozzle (Δ*P* = *P*_d_ – *P*_s_). This can be expressed in terms of the discharge coefficient, *C*_d_, a dimensionless group defined thus:

(1) $$C_{d}=\frac{4m}{\pi D_{t}^{2}\sqrt{2\rho \Delta P}}$$

Here *ρ* is the liquid density and *D*_t_ the nozzle diameter. Gordon and others (2012) reported the following relationship between the *C*_d_ and the dimensionless clearance, *h*/*D*_t_,

(2) $$C_{d}=a_{1}\left(1-e^{-a_{2}\left[\left(h/D_{t}\right)+a_{3}\right]}\right)$$

where *a*_1_, *a*_2_, and *a*_3_ are constants. By maintaining a constant mass flow rate, adjusting the clearance height and measuring Δ*P*, calibration curves may be obtained to give the constants in Eq. 2 (see Supplementary Information, Figure S3).

The shear stress exerted on the surface under the nozzle depends on the mass flow rate and clearance. For viscous flows, where the Reynolds number (defined Re = *4* $\dot{m}$/*πμD*_t_) is ≤100, the shear stress on the surface, *τ*, can be estimated from Middleman (1998),

(3) $$\tau =\frac{3\mu }{\pi }\frac{Q}{h^{2}}\frac{1}{r}$$

Here *Q* is the volumetric flow rate of liquid, *μ* its viscosity, and *r* is the radial coordinate (*r > D*_t_ /2). The maximum shear stress is found at the inner lip of the nozzle, however spores were counted over the annular area between the inner and outer radius of the nozzle rim, *r*_1_ and *r*_2_, respectively (Figure S5). The average stress evaluated over the annulus is given by,

(4) $${\bar{\tau }}_{\mathrm{wall}}=\mu \left[\frac{3Q}{4\pi {\left(h/2\right)}^{2}}\right]\frac{2}{\left(r_{1}+r_{2}\right)}$$

Testing a successive number of locations with different shear stresses yields an adhesion profile relating the fraction of spores remaining on the surface (*N*/*N*_0_) to the applied shear stress applied, evaluated using Eq. 4.

If the adhesive properties of a population of spores are normally distributed, the following relationship is expected to apply:

(5) NN0=1−12πσ2∫0τ¯ wall exp−τ−τ ave 2σ2dτ

where *τ* is the shear stress in the region of interest and *σ* is the standard deviation from the mean shear stress, *τ*_50%_, required to detach 50% of spores from a substrate. For each spore species, *τ*_50%_ and *σ* values were obtained by fitting the adhesion profile to Eq. 5. This shear stress can be converted to a normal adhesion force, *F*_ad_, using the result given by Mercier‐Bonin and others (2011) for a spherical body,

(6) F expt =τ ave R spore 21024+964R spore hA

where *h*_A_ is the separation between the spore and the substrate it is attached to, and *R*_spore_ is the spore's radius. As spores are not generally spherical in shape, *R*_spore_ is estimated from the equivalent volume of a cylinder with hemispherical ends, *viz*.

(7) R spore =4D3+6D2+L−D321/3

Here *D* is the diameter of the hemisphere and *L* is the end to end length of the body.

### Calculation of surface energy components

The surface free energy parameters for bacterial spores can be evaluated according to the van Oss–Chaudhury–Good (OCG) result:

(8) γL1+cosθ=2γS LW γL LW +γS+γL−+γS−γL+

where *θ* is the contact angle of the liquid on the lawn of spores, γ LW is the Lifshitz‐van der Waals (nonpolar) component of surface energy; $\gamma^{+}$ and $\gamma^{-}$ are the electron acceptor and electron donor subcomponents, and the subscripts (S) and (L) refer to the solid and liquid, respectively. The surface energy components for a range of liquids have been tabulated by Van Oss (2006): this leaves 3 unknowns in Eq. 8, which may be determined by measuring the contact angle of at least 3 liquids, where one of them is nonpolar. An exact solution to the set of equations could not always be found and in these cases the set of surface energy components of the solid was taken to be that which would minimize the total absolute difference between the 2 sides of Eq. 8.

### xDLVO modeling

In the extended DLVO theory Van Oss (1993), the total free energy of interaction between a particle and a flat surface, *G* ^Total^ can be written as:

(9) G Total =G LW +G EL +G AB +G Br

The equations used to calculate each interaction energy for 3 geometrical configurations can be found on Table 2. For spherical spores, the volume equivalent radius, *R*_spore_, was used. For cylinders, the length, *L*, and diameter, *D*, were used. For the flat surface geometry, the contact radius between the spore and surface was estimated using the JKR model of elastic contact (Johnson and others 1971),

(10) a3=9R spore 2W ad πE∗

where *W*_ad_ is the work of adhesion of between the spore (2) and the substrate (1) in water (subscript *w*) given by Butt and others (2005) as,

(11) W ad 2=2γw+γw−+γ1+γ2−+γ1−γ2+−γ1+γw−−γ2+γw−−γ1−γw+−γ2−γw+−γ1 LW −γw LW γw LW −γ2 LW

with *E* ^*^ the reduced Young's modulus,

(12) 1E∗=1−ν spore 2E spore +1−ν surface 2E surface

**Table 2:** van der Waals, electrical double layer and Lewis acid–base interaction energies between macroscopic bodies of 3 different geometries and surfaces

Geometry of bodies with surfaces *h*_A_ apart (*h*_A_ ≪ *R*)		Lifshitz‐van der Waals interaction energy^1^	Electrostatic double layer interaction energy^2^	Lewis acid‐base interaction energy^3^
Sphere on a flat surface	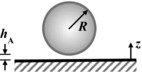	G LW =A1W2R6z	G EL =RZe−KZ	G AB =2πRλΔG1w2 AB eD0−zλ
Cylinder on a flat surface (per unit length)	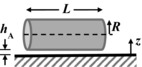	G LW =A1W2R122z3/2	G EL =K1/2R2πZe−KZ	G AB =RλΔG1w2 AB eD0−zλ
Two flat surfaces (per unit area)	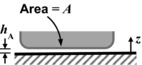	G LW =A1W212πz2	G EL =K2πZe−KZ	G AB =ΔG1w2 AB eD0−zλ

Adapted from Leckband and Israelachvili (2001) and Van Oss (2006).

1. The Hamaker constant between body 1 and surface 2 is given by Eq. (13).

2. The electrical double layer function, *Z*, is given by Eq. (14). *κ* ^−1^ is the Debye length (*κ* ^−1^ = 960 nm for pure water).

3. Equations for Lewis acid–base interaction energies can be found in Van Oss (2006). *λ* is the characteristic decay length for water taken as 1 nm (Israelachvili 2010).

Here *ν_i_* is the Poisson's ratio of material *i*, estimated at 0.3 (Zhao and others 2005). The Young's modulus of these spores was measured at approximately 2 GPa (Wang and others 2015).

For *B. megaterium* and *B. subtilis* the contact area was estimated as the product of the JKR contact radius, *a*, and the spore length, *L*; however, for *B. cereus* spores, the contact area was determined by the size of the exosporium observed in SEM images (Zhou and others 2017).

The *γ* ^LW^ term contains the Hamaker constant *A*_132_ between the spore (1) and surface (2) in water (w). This is evaluated using,

(13) A1w2=24πhA2γ1 LW −γw LW γ2 LW −γw LW

The *G* ^EL^ term contains the function

(14) $$Z=64\pi \varepsilon_{0}\varepsilon_{r}{\left(\frac{kT}{e}\right)}^{2}\tanh^{2}\left(\frac{e\psi_{a}}{4kT}\right)$$

where *ε*_0_ is the permittivity of free space (8.85 × 10^−12^ m^−3^ kg^−1^ s^4^ A^2^), *ε*_r_ is the relative permittivity (80 for water at 25 °C), *k* is the Boltzmann constant (1.38 × 10^−23^ m^2^ kg/s^2^/K), *T* is the absolute temperature and *Ψ*_a_ is the aggregate surface potential between the spore and surface. As *Ψ*_a_ cannot be determined readily it is frequently approximated by the zeta potential (Leckband and Israelachvili 2001) and the aggregate zeta potential is evaluated as the geometric mean of the zeta potentials (Van Oss 2006).

The Lewis acid–base interaction term ΔG1w2 AB  is given by,

(15) ΔG1w2 AB =2γ1+γw−+γ2+γw−+γ1−γw++γ2−γw+−2γw+γw−−γ1+γ2−−γ1−γ2+

where the surface energy components for the spore (2) and the surface (1) are evaluated using Eq. (8), and that for water (w) is taken from Van Oss (2006).

Liu and Zhao (2005) estimated the Brownian motion energy component to be 0.414 × 10^−20^ J at room temperature, which is insignificant when bacterial spores adhere.

The force between the 2 surfaces is the derivative of the total energy with respect to distance from the surface, that is,

(16) F DLVO =−dG Total dz

where *z* is the coordinate normal to the surface (see sketches in Table 2). An initial estimate of the adhesive force can therefore be obtained by assuming an universal cut‐off distance, *h*_0_, of 0.165 nm (see Israelachvili (2011)). This parameter can be adjusted for each geometry to minimize the error between the measured adhesion force calculated using Eq. (6) and the model.

## Results

### Spore size and contact area

Prior work (Zhou and others 2017) had quantified the size of the various spore samples studied here. These results, together with the equivalent spore radius and contact area are presented in Table 3. By approximating the projected area of a *B. megaterium* spore as an ellipse, the maximum contact area for “BMEG” can be estimated to be 0.82 μm^2^. On this basis, its contact area is a reasonable fraction, at 10% to 15% of the maximum contact area. Nevertheless, these values are likely to be an underestimate for the actual contact area as the effects of spore orientation and additional exosporium rim area have not been included. Spores with larger and more flexible exosporia tend to have larger contact areas.

**Table 3:** Summary of the measured and calculated dimensional parameters required in bacterial spore adhesion model

Label	Length, *L* (μm)	Diameter, *D* (μm)	Equiv. spherical radius, *R*_spore_ (μm)	Average contact radius, *a*_ave_ (μm)	Contact area (μm^2^)
*B. megaterium* QM B1551					
BMEG	1.15	0.91	0.50	0.049	0.11
BMEG25	1.17	0.89	0.49	0.055	0.12
BMEG37*	1.00	0.84	0.45	0.046	1.28
BMEGPH5.5*	1.13	0.89	0.49	0.049	1.54
BMEGPH9*	1.08	0.85	0.46	0.052	1.34
BMEG1	1.30	0.90	0.54	0.054	0.12
BMEG1,25,PH9	1.40	0.96	0.57	0.047	0.12
*B. cereus* 569*					
BCEREUS	1.54	0.85	0.55	0.046	1.81
BCEREUS25	1.51	0.83	0.54	0.052	1.85
BCEREUS37	1.44	0.78	0.51	0.044	1.35
BCEREUSPH5.5	1.53	0.80	0.54	0.046	1.74
BCEREUSPH9	1.39	0.77	0.50	0.049	1.49
BCEREUS1	1.51	0.81	0.53	0.048	2.10
BCEREUS2	1.53	0.82	0.54	0.042	1.58
BCEREUS2,PH9	1.54	0.81	0.54	0.043	1.61
*B. subtilis* PS832					
BSUBT	1.30	0.75	0.48	0.044	0.12

The equivalent spherical radius and contact area were evaluated using Eq. (7) and (10), respectively. For samples marked with an asterisk (*), the contact areas were measured using SEM images obtained from previous work

### Spore surface properties

Table 4 summarizes the measured and calculated surface properties of bacterial spores cultured under a variety of broth conditions. It is evident that most spores, apart from the 2 *B. cereus* samples cultured in a 12.5‐fold increase in inorganic salt concentration, possess highly hydrophobic surfaces, as reported previously (Rönner and others 1990; Faille and others 2002). Indeed, the contact angle of water on *B. cereus* spores is comparable to that on Teflon^®^ which is between 100° and 121°. The zeta potentials measured for *Bacillus* spores are in the same range as the range −14 and −35 mV reported by Husmark and Rönner (1992) and (Faille and others 2010) for a variety of species. When the salt concentration in the culture broth is increased, the hydrophobicity, and zeta potential of both *B. megaterium* and *B. cereus* spores decrease. This is likely to be caused by the adsorption of insoluble precipitates to the spore exosporium, affecting the spore's surface properties. Of the 16 spore samples in Table 4, 10 possess either an acidic (*γ^+^*) or basic component (*γ^–^*) alone, as adjudged from the OCG equation, suggesting that the spores’ surface tends to be monopolar. Among these monopolar samples, 6 are basic, suggesting a balance between acidic and basic surfaces.

**Table 4:** Hydrophobicity, zeta potential, and surface energy parameters of bacterial spores and substrates studied

			Surface energy parameters (mJ/m^2^)		
Label	H_2_O contact angle (°)	Zeta potential (mV)	***γ*^LW^**	***γ^+^***	***γ*^–^**
*B. megaterium* QM B1551					
BMEG	101 ± 2	−15 ± 1	29.0 ± 0.2	0.14 ± 0.02	0.2 ± 0.1
BMEG25	77 ± 7	−21 ± 1	36.7 ± 0.2	0	10 ± 1
BMEG37	98 ± 2	−15 ± 1	21.0 ± 0.2	0	3.3 ± 0.4
BMEGPH9	83 ± 2	−12 ± 1	23.2 ± 0.2	0	13 ± 1
BMEGPH5.5	98 ± 4	−22 ± 3	35.1 ± 0.5	0.3 ± 0.05	0.2 ± 0.1
BMEG1	94 ± 5	−3 ± 1	30.7 ± 0.3	0.37 ± 0.04	1 ± 0.6
BMEG1,25,PH9	117 ± 3	−9 ± 1	32.7 ± 0.4	0	0
*B. cereus* 569					
BCEREUS	109 ± 1	−26 ± 1	29.8 ± 0.4	0	0
BCEREUS25	116 ± 2	−33 ± 2	26.7 ± 0.3	0.02	0
BCEREUS37	114 ± 3	−30 ± 2	29.9 ± 0.4	0.2 ± 0.1	0
BCEREUSPH9	114 ± 2	−26 ± 1	29.0 ± 0.4	0.2 ± 0.1	0
BCEREUSPH5.5	112 ± 2	−18 ± 3	25.8 ± 0.2	0	0
BCEREUS1	100 ± 3	−21 ± 3	35.1 ± 0.4	0.3 ± 0.1	0
BCEREUS2	48 ± 2	−17 ± 3	44.0 ± 0.2	0	35 ± 1
BCEREUS2,PH9	69 ± 3	−22 ± 3	42 ± 1	0	14 ± 1
*B. subtilis* PS832					
BSUBT	68 ± 3	−36 ± 2	33 ± 1.4	0	21 ± 1.4
Substrates	** **		** **	** **	
Glass	0	−62.2^a^	43.7 ± 0.7	0.27	61
Stainless steel	44 ± 1	−57.5^b^	42.4 ± 0.2	0.38	33.2 ± 0.4

a Zeta potential value for borosilicate glass obtained from Gu (2007).

b Zeta potential value for stainless steel 316 at pH 7 from (Harimawan and others 2013).

### Bacterial spore fluid dynamic gauging

A representative selection of bacterial spore samples were produced for each spore type and subjected to fluid dynamic gauging For some samples, a proportion of the spores were observed to detach from the substrate when it was initially immersed in water. These fractions are labeled as “weakly adherent” in Table 5 and were considered to have an adhesive shear stress below the lower limit of the FDG device (approximately 20 Pa). In contrast, shear stresses exceeding 1500 Pa were incapable of removing some *B*. *cereus* variants. Table 5 summarizes the shear stress required to remove 50% of adherent spores for all samples studied with FDG; the associated detachment profiles are given in Figure S4. The Table also reports the shear stress required to remove 95% of adherent spores, where this could be reliably determined. As found by Husmark and Rönner (1992) and Faille and others (2002), spores generally adhere better to stainless steel than glass. Spores with a more hydrophobic surface (*B. cereus*) tend to display stronger adhesion those with a more hydrophilic surface (*B. subtilis*). The standard deviation is noticeably large; the coefficients of variation (that is, standard deviation − mean) in Table 5 range between 0.42 and 1.45.

**Table 5:** Summary of the adhesive properties of the bacterial spores studied

		Glass			Stainless steel		
Label	H_2_O contact angle (°)	% weakly adherent	*τ*_50%_ (*σ*) (Pa)	*τ*_95%_ (Pa)	% weakly adherent	*τ*_50%_ (*σ*) (Pa)	*τ*_95%_ (Pa)
*B. megaterium* QM B1551							
BMEG	101 ± 2	5%	52 (37)	114	1%	232 (228)	605
BMEG25	77 ± 7	52%	<20	N.D.	58%	<20	N.D.
BMEGPH9	83 ± 2	50%	<20	N.D.	33%	<20	N.D.
BMEG1,25,PH9	117 ± 3	4%	116 (49)	197	14%	226 (105)	400
*B. cereus* 569							
BCEREUS	109 ± 1	0%	>1500	N.D.	0%	>1500	N.D.
BCEREUSPH9	114 ± 2	5%	>1500	N.D.	20%	383 (356)	970
BCEREUS2	48 ± 2	38%	161 (234)	546	0%	>1500	N.D.
BCEREUS2,PH9	69 ± 3	4%	>1500	N.D.	44%	<20	N.D.
*B. subtilis* PS832							
BSUBT	68 ± 3	65%	<20	N.D.	80%	<20	N.D.

The average detachment shear stress (*τ*_50%_), level for 95% removal (*τ*_95%_), and standard deviation (*σ*) were evaluated from normal distribution fitting and the adhesion force estimated from Eq. (5). Undetermined values labeled N.D.

**Figure 1:**
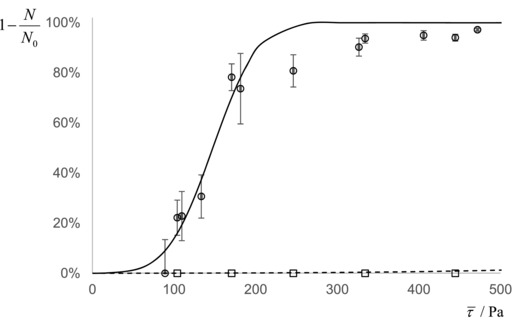
Plot showing the effect of surfactant (Tween 20) on the fraction of adherent *B. cereus* spores (‘BCEREUS’) remaining on glass after being subjected to the stated shear stress. Symbols: □, *B. cereus*; ○, *B. cereus* washed with 0.1% Tween20. Lines represent the data points fitted to the cumulative normal distribution function, Eq. (5); ─ ─, *B. Cereus* (*τ*_50%_ > 1500 Pa: data collected at *τ* > 500 Pa have not been plotted, for clarity); ──, *B. cereus* washed with 0.1% Tween20 (*τ*_50%_ = 147 Pa, *σ* = 44 Pa).

The measurements in Table 5 were made with spores subjected to a deionized water environment throughout the spore preparation steps. *B. cereus* samples, washed in the presence of 0.1 vol% Tween 20 surfactant, which is often used to minimize spore clumping, but subsequently gauged with deionized water, gave very different results. Without Tween 20, *B. cereus* spores require shear stresses greater than 1500 Pa for detachment. In the presence of Tween 20, however, Figure 1 shows this shear stress is reduced to approximately 150 Pa, indicating that hydrophobic interactions have been significantly reduced.

### Spore adhesion modeling

Table 6 reports the predicted adhesion force, *F*_DLVO_, of a single bacterial spore attached to the corresponding substrate calculated for the geometries considered. Adhesion to stainless steel is expected to be stronger than to glass due to the more hydrophobic nature of the stainless steel surface. Prior work on spore force spectroscopy indicates that the adhesion force lies within the range of 3 to 150 nN (Bowen and others 2002; Chung and others 2010; Harimawan and others 2013), suggesting that the calculated values are of the correct order of magnitude.

**Table 6:** Spore adhesion force predicted by xDLVO model for 3 different geometries

	Adhesion force,$F_{ad}^{DLVO}$, evaluated for different geometries immersed in water (nN)					
Label	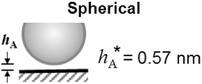		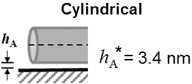		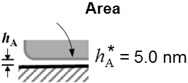	
	Glass	Stainless steel	Glass	Stainless steel	Glass	Stainless steel
*B. Megaterium*						
BMEG^*^	8 ± 2	45 ± 2	4 ± 1	20 ± 1	4 ± 1	18 ± 1
BMEG25	−18 ± 2	17 ± 2	−8 ± 1	8 ± 1	−7 ± 1	9 ± 1
BMEG37	−1 ± 1	31 ± 1	0 ± 1	13 ± 1	−6 ± 6	159 ± 6
BMEGPH9	−27 ± 2	7 ± 2	−12 ± 1	3 ± 1	−140 ± 10	36 ± 10
BMEGPH5.5	0 ± 3	38 ± 2	0 ± 1	17 ± 1	14 ± 19	229 ± 16
BMEG1	−12 ± 5	30 ± 5	−5 ± 3	14 ± 3	−4 ± 2	13 ± 2
BMEG1,25,PH9^*^	34 ± 1	74 ± 1	16 ± 1	35 ± 1	13 ± 1	28 ± 1
*B. cereus*						
BCEREUS	36 ± 1	74 ± 10	19 ± 1	38 ± 4	226± 15	450± 30
BCEREUS25	28 ± 1	66 ± 7	14 ± 1	34 ± 3	179 ± 10	416 ± 13
BCEREUS37	15 ± 6	53 ± 5	7 ± 4	26 ± 3	78 ± 35	260 ± 29
BCEREUSPH9	14 ± 4	52 ± 3	7 ± 4	25 ± 2	85 ± 27	285 ± 22
BCEREUSPH5.5	35 ± 7	72 ± 3	18 ± 4	37 ± 3	210 ± 20	426 ± 32
BCEREUS1	8 ± 3	49 ± 2	4 ± 4	25 ± 1	77 ± 26	367 ± 21
BCEREUS2	−66 ± 1	−29 ± 1	−38 ± 1	−17 ± 1	−325 ± 14	−118 ± 8
BCEREUS2,PH9	−28 ± 2	10 ± 2	−15 ± 1	5 ± 1	−133 ± 16	74 ± 15
*B. subtilis*						
BSUBT	−39 ± 2	−5 ± 2	−18 ± 1	−2 ± 1	−16 ± 1	−1 ± 1

The cut‐off distance, *h*_A_, was determined by minimizing the difference between *F*_expt_, the experimentally determined shear stress converted to an adhesion force by Eq. (6), and *F*_DLVO_ calculated from Eq. (16) for the species labeled with an asterisk, BMEG and BMEG1,25,PH9

**Figure 2:**
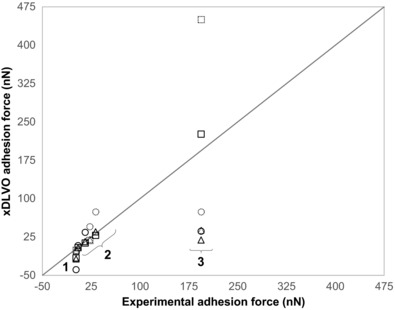
Comparison of experimentally extracted adhesion forces and those estimated by the xDLVO model in 3 different geometrical configurations. The numbers label spore species: (1) *B. subtilis* spores (“BSUBT”); (2) *B. megaterium* spores (“BMEG” and “BMEG1,25,PH9”); (3) *B. cereus* spores (“BCEREUS”). The dashed locus shows the line of equality, *y* = *x*. Symbols: *F*_DLVO_; ○, spherical model; △, cylindrical model; □, plate model; solid symbols, glass; dashed, stainless steel.

**Figure 3:**
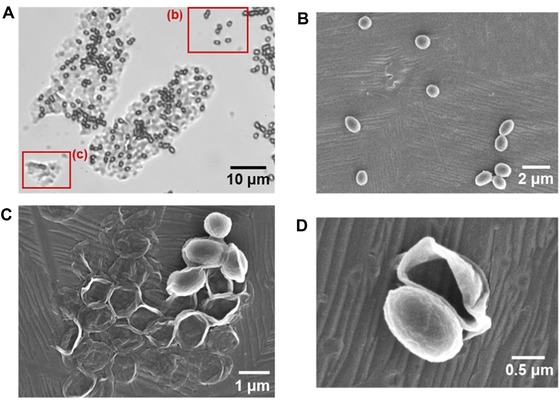
Optical microscopy and SEM images of the spores that remain attached after FDG testing. (A) 1000 × bright field optical microscopy image of *B. cereus* spores that remain attached to glass. B and C are regions of individual and smaller groups of spores attaching; (B) SEM of individual *B. megaterium* spores that remain attached to stainless steel after gauging, similar to B—the spores seem to have lost the exosporium; (C) smaller group of *B. megaterium* spores similar to C—exosporium skin is left attached to stainless steel; (D) individual *B. megaterium* spore core, showing the exosporium having been removed by the forces exerted during gauging.

Comparing the predicted and measured adhesion forces, Figure 2 indicates that the 3 geometries modeled are able to describe the adhesion of *B. megaterium* and *B. subtilis* spores. For *B. cereus*, however, the presence of a large exosporium results in significant underestimation in the spherical and cylindrical models and therefore the “plate” geometry gives a better description of this increased adhesion strength. Although the predicted adhesion force of the plate geometry for *B. cereus* to stainless steel exceeds the measured force, it should be noted that an actual measurement was not made; the measured forces were approximated using the maximum FDG shear stress of 1500 Pa for both stainless steel and glass substrates. Optical microscopy images such as Figure 3(A) were often obtained when inspecting gauging locations. The identity of the translucent structure in this image was uncertain but subsequent SEM analyses (Figure 3B–D) revealed it to comprise exosporia, presumably shed from spores by shear stress forces exerted during gauging.

### Surface properties of germinating spores

**Figure 4:**
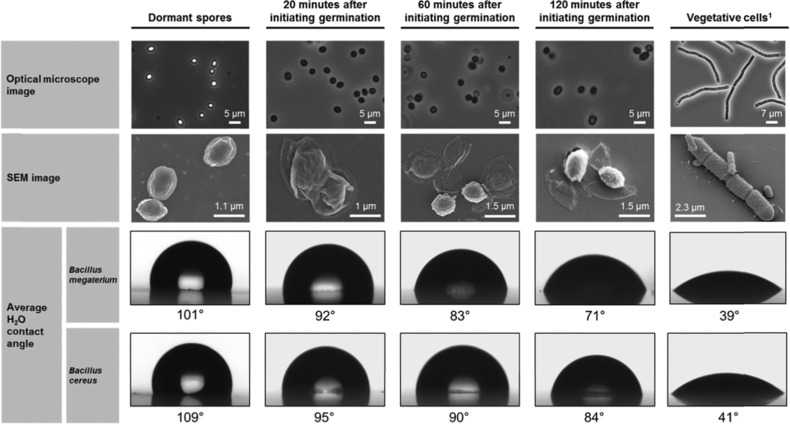
Phase contrast optical microscopy and SEM images showing the appearance of *B. megaterium* QM B1551 spores as they transition from dormant spores to vegetative cells. The average contact angle of water measured for each sample and species shows the transition from a hydrophobic spore to a hydrophilic vegetative cell. 1SEM image for B. megaterium QM B1551 vegetative cell taken from Vary and others (2007)

The SEM images in Figure 4 show the change in morphology during the germination of *B. megaterium* spores. In the 1st 30 min, most spores retain their original structure with the central spore integuments contained within the exosporium. The size of the spore has increased considerably primarily as a result of water uptake (Setlow 2003). As germination progresses, the germinated spore is released from the exosporium and elongates, becoming a vegetative cell. The hydrophobicity of the spore surface (that is, the exosporium) decreases as germination progresses. For both *B. megaterium* and *B. cereus*, the contact angle of water decreased from over 100° for dormant spores to around 40° for vegetative cells.

In addition to hydrophobicity, Table 7 shows that the zeta potential and surface energy components also change in well‐defined trends. For both species, γ LW  exhibits a large initial increase and tends slowly thereafter towards the vegetative cell value. $\gamma^{-}$(electron donation) increases steadily for both *B. megaterium* and *B. cereus* whereas $\gamma^{+}$ remains at 0. Collectively these results indicate that the surface of the spore is continuously changing as it develops into a new vegetative cell. Zeta potential measurements show that for the strains of *B. megaterium* and *B. cereus* used here, vegetative cells possess a more negatively charged surface than spores. This does not hold for all *Bacilli*, because Ahimou *et al*. (2001) reported that the zeta potentials in 9 *B. subtilis* strains did not vary systematically according to their state. During germination, however, a systematic change in zeta potentials may be observed, particularly throughout the 1st 20 min. For *B. megaterium*, the potential decreases from −15 to −23 mV whereas for *B. cereus* it increases slightly from −26 to −22 mV. Collectively, these results indicate that the exosporium physical properties change during germination.

**Table 7:** Zeta potential and surface energy parameters calculated for bacterial spores, vegetative cells, and at various time intervals after triggering germination

		Surface energy parameters (mJ/m^2^)		
Label	Zeta potential (mV)	*γ* ^+^	*γ^−^*	*γ* ^LW^
*B. megaterium*	* *	* *	* *	* *
BMEG	−15 ± 1	0.14 ± 0.02	0.2 ± 0.1	29.0 ± 0.2
BMEGT20	−23 ± 2	0.03	2.2 ± 0.4	32.6 ± 0.2
BMEGT60	−30 ± 3	0.03	6 ± 0.7	33.3 ± 0.9
BMEGT120	−32 ± 3	0	17 ± 0.6	34.7 ± 0.5
Vegetative cells	−57 ± 2	0	56 ± 3	33.0 ± 2.7
*B. cereus*	* *	* *	* *	* *
BCEREUS	−26 ± 1	0	0	29.8 ± 0.4
BCEREUST20	−22 ± 2	0.2 ± 0.1	0.8 ± 0.1	32.0 ± 0.2
BCEREUST60	−38 ± 4	0	3.3 ± 1	34.0 ± 0.4
BCEREUST120	−32 ± 7	0	5.7 ± 1	36.2 ± 1.1
Vegetative cells	−40 ± 3	0	50 ± 4	36.5 ± 0.5

**Figure 5:**
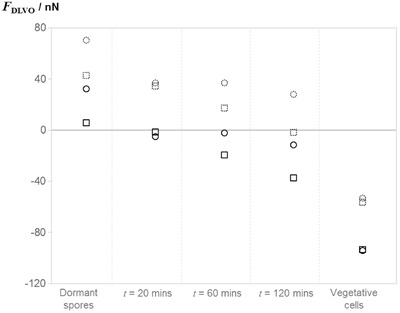
Adhesion force to stainless steel 316 and glass surfaces predicted by the xDLVO model, *F*_DLVO_, for spores at various times after triggering germination and vegetative cells. Symbols show; □, *B. megaterium*; ○, *B. cereus*: solid symbols, glass; dashed, stainless steel.

The surface energy components and zeta potential measurements from Table 7 were then used to predict the force of adhesion for spores undergoing germination. Figure 5 shows how the adhesion force changes for spores at different stages during germination. The spores were assumed to be spherical with constant size; although the images in Figure 4 shows that neither assumption is completely correct, the model allows the changes in the spore surface characteristics to be quantified. Triggering germination significantly reduces the force of adhesion in both *B. megaterium* and *B. cereus*. For instance, 20 min after initiating germination in *B. megaterium*, the predicted adhesion force on glass is effectively 0. This agrees with phase contrast microscopy testing: as the firmly adhered phase‐bright spores turn dark, many detach spontaneously as a result of Brownian forces.

## Discussion

The adhesive strength of spores of several *Bacillus* species has been quantified. Table 5 shows that the average shear stress required to detach spores is an order of magnitude higher than that required for vegetative cells (around 10 Pa, Powell and Slater 1982). Moreover, these values lie beyond the shear stresses that are achieved in standard cleaning‐in‐place (CIP) protocols (approximately 5 Pa) and would require more advanced technology, such as rotating jet heads for tank cleaning, that can deliver shear stresses in the range of 50 to 1000 Pa (Jensen and others 2012). *B. cereus* spores cultured under standard conditions exemplify the strength of adhesion, where shear stresses of order 1500 Pa were unable to disrupt adherent spores. In addition, there was no visual evidence of disruption to the layer of deposited spores. The result was confirmed qualitatively by withdrawing *B. cereus* samples from the FDG rig and subjecting them to a vigorous impinging jet of liquid. The samples remained intact, with no visual evidence of damage to the layer of spores.

Mercier‐Bonin and others (2011) employed a parallel plate flow chamber to study the adhesion of several *Bacillus* spore species to stainless steel and their results share many similarities with Table 5. They observed no significant detachment for 2 samples of spores, *B. cereus* 5832 and *B. thuringiensis* 407, for *τ* ≤ 80 Pa. Two other types of spore, *B. cereus* 98/4, and *B. pumilus* 98/6, exhibited a wide distribution in adhesion strengths (similar to Figure S4) but were less adherent, with an average detachment shear stress, *τ*_50%_, of 24.8 ± 5.5 and 53.8 ± 9.1 Pa, respectively. The large variance in adhesion strength is likely to be another contributor to the difficulties in cleaning *B. cereus* spores, in addition to their resistance to chemical and environmental stress. This variability could arise from different orientations or substrate topographies, but it is equally likely that variability within spore populations arises from stochastic factors (perhaps at the genetic regulatory level). Such variability is also reported for spore germination (Zhang and others 2010), suggesting that variance within populations acts as a strategy for survival.

The trends observed in adhesion strength as a function of spore hydrophobicity are in agreement with prior work by Faille and others (2002) and (Rönner and others 1990). The average detachment shear stresses in Table 5 show that all spores generally adhere better to a more hydrophobic surface. Across species, those possessing a more hydrophobic surface (for example, *B. cereus*) adhere more strongly than those with a hydrophilic surface (*B. subtilis*). This trend is also evident for spores of the same strain, cultured under different conditions. This contrasts with the findings of Tauveron and others (2006) and Faille and others (2010) where the strong influence of hydrophobicity was only found to apply when considering spores across a variety of species, and was unable to fully explain the differences in adhesion of multiple strains within a particular species. One possible explanation for this could be differences in methodologies employed to quantify hydrophobicity. However, it is perhaps more feasible that their use of ultra‐sonication introduced a bias to the force experienced by a spore, because larger spores would experience a greater force. This could potentially explain the conclusion reached by Tauveron and others (2006), who reported that *B. cereus* spores with larger exosporia exhibited a lower resistance to cleaning.

There was one anomalous result deviating from the above trend. Culturing spores in alkaline media, whether as the single parameter changed or in combination with others, decreased their ability to adhere to stainless steel. In the case of *B. megaterium*, spores cultured in a combination of changes (5‐fold enhanced inorganic salt concentration, lower temperature and increased alkalinity), the increase in adhesion was lower than expected, whereas the 2 *B. cereus* samples cultured in enhanced alkaline conditions showed a marked reduction in adhesion. It is possible that the high pH induced changes in spore physical properties that were not captured with the measurements conducted here.

Industrial cleaning fluids are unlikely to consist solely of water. Bobe and others (2007) reported that the use of surfactants or sodium hydroxide solutions reduced the detachment force of spherical polystyrene particles by a factor of 5. In this study, washing *B. cereus* spores in a solution of 0.1% Tween 20—often practiced by researchers in this field—reduced their adhesion strength by at least a factor of 10. This augmented surfactant effect is considered to arise from the spore adhesion being strongly dependent on hydrophobic interactions. FDG measurements nevertheless showed that surfactant‐washed *B. cereus* spores adhered with considerable strength to glass (*τ*_50%_ ≈ 147 Pa), which is consistent with Rönner and Husmark (1992)’s finding that approximately 60% of *B. cereus* spores remained on stainless steel coupons despite extensive CIP steps.

The errors shown in Table 6 arise from the uncertainties in contact angle measurements. The magnitude of these errors suggest that the method can produce reasonably reliable predictions for adhesion force. Notwithstanding, there are 3 parameters within the model that can significantly affect the predicted force, namely the clearance between the particle and the surface, *h*_A_; the characteristic decay length of water, *λ*; and the size parameters for the spore (radius, contact area, and so on).

Oliveira (1997) reported a characteristic water decay length of 0.2 nm for pure water. Given the readiness with which ions dissolve in water, the ionic strength in the gauging liquid is expected to be non‐zero and will increase *λ*, closer to 1 nm as suggested by Leckband and Israelachvili (2001).

The clearance also has a significant impact on the xDLVO prediction. A clearance of 0.66 nm for the spherical geometry seemed low, especially considering that spore surface features can be of order 10 nm (Faille and others 2010). Mercier‐Bonin and others (2011) associated the clearance distance with the length of the spore's filaments (25 nm) and Vigeant and others (2002) suggested that the clearance would be of the order of 10 nm for irreversibly adhering cells. The distances determined for the cylindrical and plate models are more reasonable estimates of *h*_A_. In practice, however, it is likely that each sample tested by FDG in Table 5 would have its own characteristic separation distance, which may explain some of the aforementioned anomalies. Furthermore, within a population, there may be a wide distribution of *h*_A_ values, which may contribute to the breadth of the removal profiles in Figure S4.

Although Figure 2 shows that the xDLVO model gives reasonable predictions of the adhesion strength for *B. megaterium* and *B. subtilis* spores, it also shows that the *B. cereus* exosporium size must be considered. Indeed, Figure 3 shows that the forces imposed in FDG testing are large enough to remove the spore core from its exosporium, suggesting that the exosporium may have a stronger role in spore adhesion than spore protection or germination. The loose and flexible exosporium in *B. cereus* spores not only increases the contact area with the substrate, but also alters the fluid flow pattern around the particle, reducing the effectiveness of flow in detachment. Pozrikidis (1997) determined the flow pattern over a hemisphere and showed that the forces experienced by this shape are approximately half those of a sphere. Given that many of the spores studied are nonspherical, most of the adhesion forces determined may be over‐estimates. Other factors that should also be considered include substrate topography and roughness.

The observed and predicted reduction in adhesion force during germination is significant for food processing. One strategy that could improve cleaning efficiency for bacterial spores would involve adding a trace of germinant to the cleaning fluid. This simple step requires further investigation, as not all spores respond to the same germinant and they are mostly asynchronous in their germination process.

## Conclusions

Shear flow testing using the fluid dynamic gauging technique has provided quantitative estimates of the adhesion of bacterial spores to glass and stainless steel. *B. cereus* required shear stresses significantly higher than those imposed in standard CIP operations. FDG testing allows multiple measurements to be made on the same surface, which allowed the distribution of spore adhesion forces to be identified and quantified.

The adhesion force, which could be related to the size and hydrophobic nature of the exosporium, varied between species. Variations in temperature, initial pH and inorganic salt levels during spore formation affected the resultant spore surface properties and their adhesion behavior. Each spore population exhibited a wide distribution of adhesion strengths.

The adhesion force was modeled using the xDLVO approach. The model could describe the trends observed between spore species and variants adequately, particularly when the cut‐off distance was allowed to vary between geometries. It could not, however, predict the observed width of distribution of adhesion forces, partly because contact angle measurements were unable to capture the differences in all the factors that affect spore adhesion.

## Notation

**Table:** 

Roman		
*A*	Area	m^2^
*a*	Contact radius	m
*A*_1w2_	Hamaker constant between surfaces 1 and 2 in water	J
*C*_d_	Discharge coefficient	–
*D*	Diameter	m
*D*_t_	Nozzle diameter	m
*e*	Electron charge	C
*E*	Young's modulus	Pa
*F*	Force	N
*G*	DLVO interaction energy	J
*h*	Clearance	m
*h*_0_	Universal cut‐off separation distance (0.165 nm)	m
*k*	Boltzmann constant	J/K
*L*	Length	m
*m*	Mass flow rate	g/s
*N*	Spore coverage after gauging	m^−2^
*N*_0_	Initial spore coverage	m^−2^
*P*	Pressure	Pa
*Q*	Volumetric liquid flow rate	m^3^/s
*r*	Radial distance	m
*R*	Sphere radius	m
*T*	Absolute temperature	K
*W*_ad_	Work of adhesion	J/m^2^
*z*	Perpendicular distance from a flat surface	m
**Greek**		
*γ*	Surface energy	J/m^2^
*θ*	Liquid contact angle	°
*κ* ^−1^	Debye length	m
*λ*	Water decay length, ∼1 nm	m
*μ*	Viscosity	Pa s
*ν*	Poisson's ratio	–
*ρ*	Density	kg/m^3^
*σ*	Standard deviation of the mean	–
*τ*	Wall shear stress	Pa
*ψ*_a_	Aggregate surface potential	V
		

## Authors’ Contributions

K. Xu Zhou designed and performed the experiments and drafted the manuscript, N. Li built the hard disk spin coater, automated and performed the data analysis, G. Christie and D. I. Wilson are both corresponding authors who provided guidance with the experimental design, interpretation of results, and revision of the manuscript.

## Supporting information

Table S1–Concentration of culture broth nutrients and salts used to form spores of each respective *Bacillus* species.
Figure S1–FDG sample preparation procedure.
Figure S2–Schematic of the FDG nozzle section showing the fluid flow profile which exerts shear stress, *τ*, on a sample of spherical particles deposited on a substrate.
Figure S3–Example of calibration curve for pressure mode FDG in ejection.
Figure S4–Effect of shear stress imposed on the surface on the fraction of adherent spores that remain.
Figure S5–Phase contrast image (100× magnification) showing a typical footprint at a gauging location.
