# Supplementary material for: Assessing the Impact of Germination and Sporulation Conditions on the Adhesion of Bacillus Spores to Glass and Stainless Steel by Fluid Dynamic Gauging
Source: J Food Sci. 2017 Nov 10;82(11):2614–25. doi: 10.1111/1750-3841.13940 (PMC5698761; doi:10.1111/1750-3841.13940)
Supplement: Supplementary file 1 — Table S1–Concentration of culture broth nutrients and salts used to form spores of each respective Bacillus species. Figure S1–FDG sample preparation procedure. Figure S2–Schematic of the FDG nozzle section showing the fluid flow profile which exerts shear stress, τ, on a sample of spherical particles deposited on a substrate. Figure S3–Example of calibration curve for pressure mode FDG in ejection. Figure S4–Effect of shear stress imposed on the surface on the fraction of adherent spores that remain. Figure S5–Phase contrast image (100× magnification) showing a typical footprint at a gauging location. [file JFDS-82-2614-s001.docx]

# Supplementary information

Table S1 – Concentration of culture broth nutrients and salts used to form spores of each respective *Bacillus* species.

| **Broth content** | ***B. megaterium*** | |  | ***B. cereus*** | |  | ***B. subtilis*** | |
| --- | --- | --- | --- | --- | --- | --- | --- | --- |
|  | Ingredient | Grams per litre |  | Ingredient | Grams per litre |  | Ingredient | Grams per litre |
| **Nutrients** | | | | | | | | |
|  | Nutrient broth (DIFCO) | 8.0 |  | Casein enzymatic hydrolysate | 1.0 |  | Agar | 17 |
|  | Glucose | 1.0 |  | Casein hydrolysate (ACID) | 1.0 |  | Peptone | 10 |
|  |  |  |  | Glycerol | 0.6 |  | Beef extract | 6.0 |
|  |  |  |  | Yeast extract | 0.4 |  | Glucose | 1.54 |
|  |  |  |  | Glutamine | 0.04 |  |  |  |
| **Salts** | | | | | | | | |
|  | KCl | 1.0 |  | KH_2_PO_4_ | 1.77 |  | KCl | 2.0 |
|  | MnCl_2_•4H_2_O | 0.004 |  | K_2_HPO_4_ | 5.93 |  | MgSO_4_•7H_2_O | 0.5 |
|  | FeSO_4_•7H_2_O | 0.0003 |  | MgCl_2_•6H_2_O | 0.102 |  | Ca(NO_3_) _2_•4H_2_O | 0.236 |
|  | MgSO_4_•7H_2_O | 0.247 |  | MnCl_2_•4H_2_O | 0.002 |  | MnCl_2_•4H_2_O | 0.0198 |
|  | CaCl_2_•6H_2_O | 0.147 |  | FeCl_3_•6H_2_O | 0.0135 |  | FeSO_4_•7H_2_O | 0.0003 |
|  |  |  |  | ZnCl_2_ | 0.0068 |  |  |  |
|  |  |  |  | CaCl_2_•6 H_2_O | 0.0294 |  |  |  |


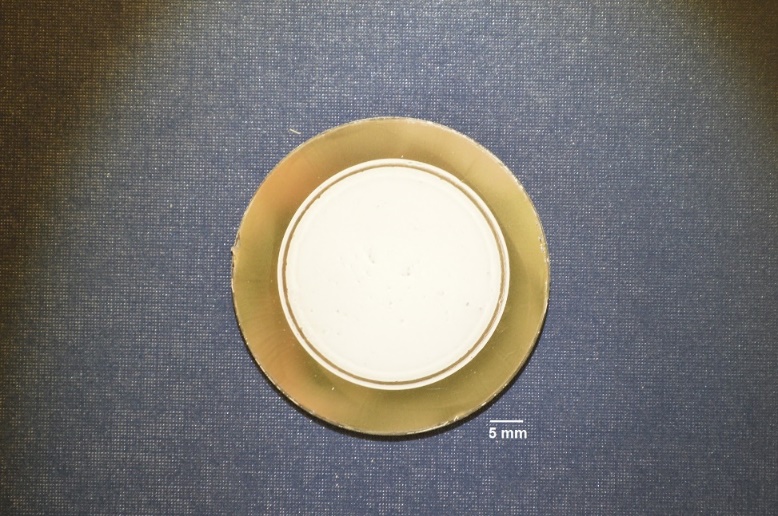

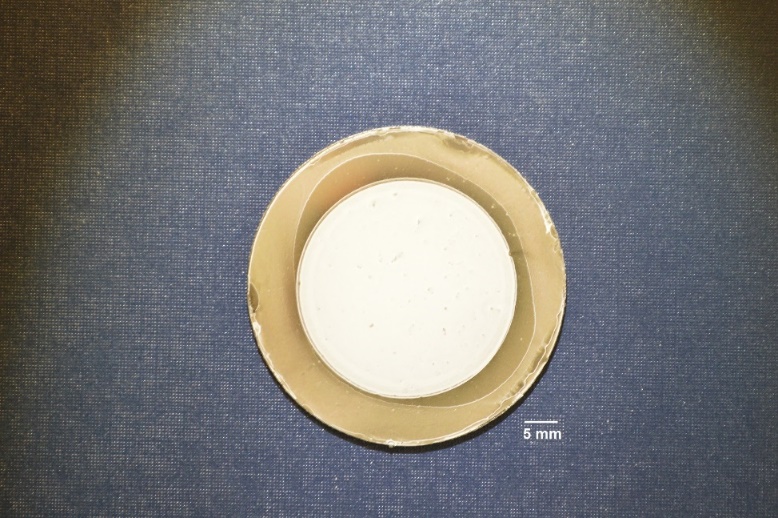


**(a)**

**(b)**

H

B

D

S

5 mm

5 mm

Figure S1 – FDG sample preparation procedure. (a), schematic of the apparatus set-up: B – spin coater base; D – hard disc drive; H – hair drier operated at low heat (< 40°C) and S – sample; (b), images of two stainless steel coupons coated with *B. megaterium* spores.

*h*

*D_t_*

*P_D_*

*P_S_*

6 mm

4 mm

*w*

*τ*

N

S

B

Figure S2 – Schematic of the FDG nozzle section showing the fluid flow profile which exerts shear stress, *τ*, on a sample of spherical particles deposited on a substrate. The adjustable parameters are mass flow rate, *m,* clearance, *h*; the nozzle diameter, *D*_t_ = 1 mm; the lip width, *w* = 0.5 mm; the dynamic pressure, *P*_D_, and static pressure, *P*_S_, are measured using pressure transducers. N – nozzle; S – deposited spheres; B – substrate

h/d_t_

*C*_d_

Figure S3 – Example of calibration curve for pressure mode FDG in ejection. The gauging liquid is water at 20°C flowing at 0.6 g s^-1^, nozzle geometry in Figure S2. ○ - experimental data; dashed line - equation (2) fitted to experimental data with *a*_1_ = 0.758, *a*_2_ = 7.18 , *a_3_* =0.0; solid line calibration curve obtained by [Salley *et al.* (2012)](#_ENREF_32) for FDG in ejection mode and nozzle dimensions *D_t_* = 1.0 mm, *w* = 0.5 mm.


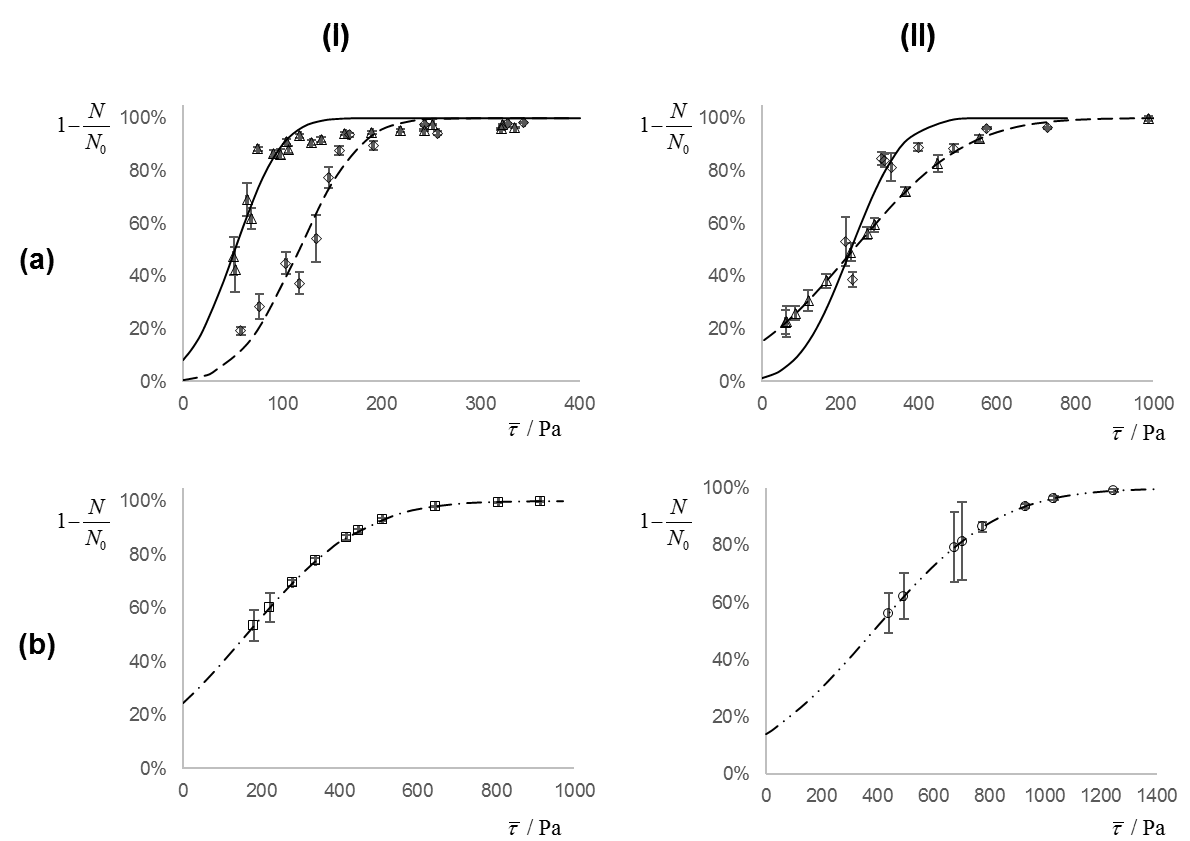


Figure S4 – Effect of shear stress imposed on the surface on the fraction of adherent spores that remain. (a) – *B. megaterium* spores; (b) – *B. cereus* spores; (i) glass substrates; (ii) stainless steel 316 substrates. Symbols: △ - BMEG; ◇ - BMEG1,25,PH9; □ - BCEREUS2; ○ - BCEREUSPH9. Lines represent the data points fitted to the cumulative normal distribution function, equation (5); ── BMEG; ─ ─ BMEG1,25,PH9; ─ • ─ BCEREUS2; ─ • • ─ BCEREUSPH9.


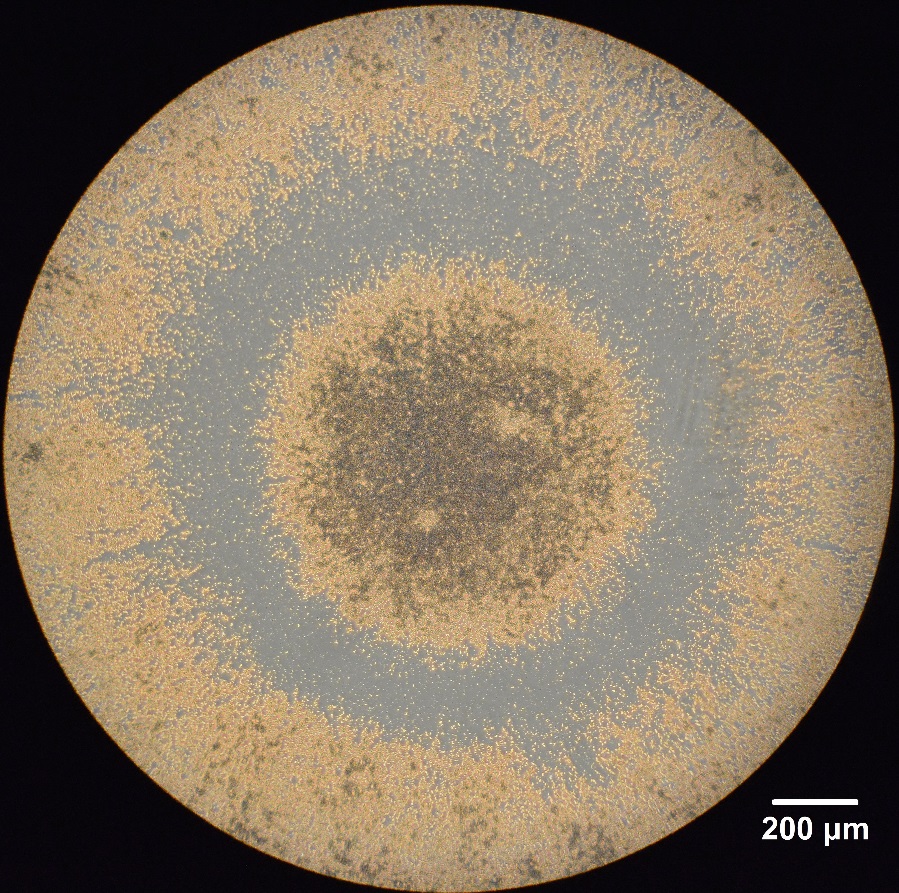


*r*_1_

*r*_2_

Figure S5 – Phase contrast image (100× magnification) showing a typical footprint at a gauging location. The area within the solid line (──) represents the nozzle throat region (diameter *D*_t_); the area between the solid and dashed (─ ─) line, at radii *r*_1_ and *r*_2_, indicates the region considered for spore counting analysis; the area between the solid and dash dot (─ • ─) line represents the nozzle lip region (*w*).
